# Experiences of advanced psychiatric nurses regarding the need for prescriptive authority in KwaZulu-Natal

**DOI:** 10.4102/hsag.v26i0.1678

**Published:** 2021-12-06

**Authors:** Eve P. Jacobs, Sipho W. Mkhize

**Affiliations:** 1 School of Nursing and Public Health, College of Health Sciences, University of KwaZulu-Natal, Durban, South Africa

**Keywords:** advanced psychiatric nurses, prescriptive authority, prescriptive gaps, prescribing, prescribing role

## Abstract

**Background:**

Nurse prescribing has become a global and transformational practice to ensure the achieving of optimal health outcomes, including advanced psychiatric nurses. Despite the transformational practice globally, South Africa seems to lag behind because nurses do not have permission to prescribe medication.

**Aim:**

To describe the experiences of advanced psychiatric nurses regarding the need to prescribe medication treatment in KwaZulu-Natal.

**Setting:**

The study took place in three mental healthcare institutions in KwaZulu-Natal in inpatient units.

**Method:**

The qualitative, descriptive design was used to collect the experiences of advanced psychiatric nurses in KwaZulu-Natal regarding the need for prescriptive authority. Six focus group interviews were conducted to gather information. The seven steps of Colaizzi’s method were used to analyze the data.

**Results:**

The study found two primary themes and two sub-themes. The findings highlighted the necessity for advanced psychiatric nurse role recognition and prescribing. Insufficient use of skilled psychiatric nurses caused delays in addressing mental health patients in emergencies.

**Conclusion:**

The two themes, prescribing role of advanced psychiatric nurses and role recognition, revealed that granting advanced psychiatric nurses’ autonomy to prescribe remained a challenge. Advanced psychiatric nurses are expected to provide high-quality care, but they are limited in their abilities. Because advanced psychiatric nurses are not used to prescribe in KwaZulu-Natal, they rely on psychiatrists to manage psychotic patients.

**Contribution:**

The evaluation of policies and procedures that guide advanced psychiatric nurses in prescribing psychotropic medications.

## Introduction

Nurses’ prescriptive authority can assist in modernising the healthcare system (Bryant-Lukosius et al. 2017:5). Nurses adopt a holistic approach to care and promote collaborative practices throughout healthcare teams (Bryant-Lukosius et al. 2017:5; Eriksson et al. 2016:5). The traditional roles of advanced psychiatric nurses have changed over the decades to fulfil certain roles performed by psychiatrists (De Nesnera & Allen 2016:482). According to Fong, Buckley and Cashin (2015:100), in countries such as the United Kingdom, Australia, New Zealand and Canada, advanced psychiatric nurses are ensuring safe and effective prescribing. This is despite the resistance of doctors to accept non-medical practitioners in prescribing.

According to Maier (2019:1), the right to prescribe medication has always been solely for the medical profession. However, this ideal has changed, because of the advancement in nurse education and training, to allow an authority to prescribe (Bellaguarda, Nelson & Caravaca-Morera 2015:1066; Graham-Clarke et al. 2019:2; Maier 2019:1). The American Association of Nurse Practitioners (AANP) recognises prescriptive authority as a scope of practice of an advanced nurse. The AANP positions itself that prescriptive authority should be uniquely authorised and regulated by nursing boards in alliance with advanced nursing roles, certification and education (AANP 2020:1). Globally, nursing roles in medication prescription have seen a remarkable increase, which, according to the International Council of Nurses (ICN), forms part of innovative fundamentals contributing to advanced practice (Guest Editorial 2014:1071). This concurs with the AANP (2015:1) who, in support of the above, stated that prescribing medication for mental healthcare users formed a fundamental and integral element of the role of advanced psychiatric nurses. This forms the legal privilege of advanced practice nurses in prescriptive authority and further demarcates the difference in scope of practice for a basic registered nurse from that of an advanced nurse (Fong et al. 2015:100).

According to Hemingway and Ely (2009:24), the nurse’s role in prescribing medications was introduced as early as 2001 within the United Kingdom, with evidence of massive global development concerning prescriptive authority for nurses in states such as the United States, Canada, Sweden, and Australia (Hemingway & Ely 2009:31). Dong-Lan et al. (2018:73) acknowledged that the aspect surrounding nurse prescribing entailed a long process of development. Despite this, results are seeing the implementation of prescriptive authority in different states at different times. This shows that there has been much growth in the area of advanced nurses’ prescriptive authority (Bellaguarda et al. 2015:1070).

Despite the literature evidenced above, there is no practice or recognition of South Africa’s prescriptive authority as a fundamental role for nurses. Nurses who have completed and attained a qualification in advanced psychiatry have no authority to prescribe medication (Schober 2016:51). This has been the case despite the findings of Docrat et al. (2019:717) who pointed out that with the decreased number of psychiatrists working in the South African public sector, it is unlikely there will be a sufficient quantity of psychiatrists available to meet the service needs of the mental healthcare users. This evident shortage of psychiatrists has had its impact by negatively influencing service delivery in rural settings within South Africa. Nurses who are employed in rural settings of South Africa do prescribe, although there is no authorisation in prescriptive authority. Nurses prescribing psychotropic drugs is to address the skills shortage that encompasses mental health, and provides the opportunity for the needs of the mental healthcare users to be met (De Kock & Pillay 2017:4).

Agbedia (2012:226) stated that because of a demanding healthcare system, swift changes are necessary for nurses to be able to deliver care. This means that nurses need to acquire the necessary skills and have sound clinical judgment and competencies that would promote and enhance the facilitation of autonomous or independent practice. This coincides with the recommendations made by the ICN, that a prescriber should have specialised knowledge and clinical experience and should meet all the requirements to prescribe (Guest Editorial 2014:1071).

Whilst various states acknowledge the independent role of advanced psychiatric nurses and their role in prescriptive authority, there has been no move to acknowledge the same within South Africa. The resultant feeling is that advanced psychiatric nurses in South Africa have no recognition to perform this role. Similarly, in KwaZulu-Natal there is no literature evidence available that supports or indicates the use of advanced psychiatric nurses in the prescribing role. Literature evidence reveals that advanced psychiatric nurses in KwaZulu-Natal have to deal with a complex milieu of mental healthcare users, as well as having the responsibility of carrying out administrative, clerical and supervisory duties (Bhagwan & Joubert 2018:49).

### Problem statement

The *Nursing Act* (Act 33 of 2005 [SA 2005]) and the *Medicines Related Substance Control Act* (Act 101 of 1965 [SA 1965]) do not permit advanced psychiatric nurses in South Africa to prescribe psychotropic drug; this is despite the evidence of the shortage of specialists in mental healthcare facilities. According to De Kock and Pillay (2017:4), there are no policies or legislation permitting psychiatric nurses to prescribe medication for mental healthcare users. Regardless of this, they are still utilised to prescribe medication in the rural areas. Advanced psychiatric nurses face severe challenges that hinder them from utilising their speciality in KwaZulu-Natal. One of those challenges is that there is poor understanding of their field and the roles they need to fulfil. The severe shortage of psychiatrists in KwaZulu-Natal remains a huge challenge, and the impact of this is that even in emergency psychiatric situations, advanced psychiatric nurses have to rely on doctors to initiate treatment and prescribe medication for mental healthcare users.

### Purpose of study

This article aimed to give a critical analysis of the experience of advanced psychiatric nurses in KwaZulu-Natal, South Africa, with respect to the need for obtaining prescriptive authority in their area of practice.

### Objective

To describe the experiences of advanced psychiatric nurses regarding prescriptive gaps and the need to obtain the permission for prescriptive authority.

## Research methodology

### Study design

The use of a qualitative descriptive design, as explained by Kim, Sefcik and Bradway (2017:1), was to describe the experiences of advanced psychiatric nurses regarding the need for prescriptive authority in KwaZulu-Natal, South Africa. The selected research design allowed the researcher to obtain quality data from the participants. In addition, the research design increased the understanding of the critical perspectives of prescriptive authority gaps as shared by the participants.

### Study setting

This study took place in the inpatient units of three mental healthcare institutions in KwaZulu-Natal. The researcher identified that these settings utilised the services of advanced psychiatric nurses.

### Study population and sampling

The study took place in the uMsunduzi and Midlands area of KwaZulu-Natal, South Africa, because of the diverse mental health services offered. The study population included advanced psychiatric nurses working in the mental healthcare institutions. The advanced psychiatric nurses in the three mental healthcare settings were selected using a non-probability sampling technique. Showkat and Parveen (2017:6) indicated that non-probability sampling generated valuable insights regarding the study phenomena. According to Creswell and Guetterman (2020:240), intentional selection of participants ensures the full understanding of the central phenomena of the study. The advantage of this sampling was that the researcher was able to select the participants purposefully based on their ability to answer the research questions. Twenty-seven male and female advanced psychiatric nurses formed part of the study because they had worked for a period of 4 years and above in the mental healthcare institutions (Table 1). These advanced psychiatric nurses had all successfully completed and obtained the post basic advanced psychiatric nursing certificate.

**TABLE 1:** Demographic data of advanced psychiatric nurses.

Age in groups	Number (*N*)	Females	Males	Years of experience
26–35	7	5	2	7 = 4+
36–45	8	3	5	8 = 20+
46–55	9	8	1	9 = 30+
56–61+	3	2	1	3 = 40+

*Source:* Jacobs and Mkhize (2021) unpublished PhD thesis

Two themes and two sub-themes emerged from the research study.

### Data collection

The use of focus group interviews was to collect data from the advanced psychiatric nurses. Creswell and Guetterman (2020:253) articulated that focus group interviews involved a process through which there were interviews conducted with a group of between four and six people. The focus group discussions involved 27 advanced psychiatric nurses from the three mental healthcare institutions. There were six focus group interviews, consisting of four to five participants in each group. To ensure anonymity, the group members were all assigned pseudonyms. Duration of the focus groups was between 40 min and 45 min, which allowed the researcher to ask questions, probe further and obtain clarity on the information shared by the participants. The participants gave permission to use an audio-recorder during data collection, and field notes were taken to gather all the information about the study. Data saturation occurred when there was no new information generated from the participants.

### Measures to ensure trustworthiness

The implementation of Lincoln and Guba’s (1985) strategies, cited by Forero et al. (2018:3), were to ensure trustworthiness.

Credibility: Prolonged engagement was through a long duration of time spent with participants. The inclusion of voluntary participants and by encouraging open communication ensured honesty of participation. Member checking ensured the clarity of findings. Data collection continued until saturation was reached, and reflective summaries after each focus group discussion were kept to engage with collected data.

Dependability: The researcher ensured dependability through careful conceptualisation of the study, data collection, analysis of data, and interpretation of the reported findings.

Confirmability: A developed audit trail included a systematic collection of all documentation, such as a reflective diary and word-for-word transcriptions. The audit trail enabled the drawing of conclusions about the collated and analysed data by an independent auditor.

Transferability was ensured by providing a detailed explanation of the context.

### Data analysis

Data analysis was carried out by using the rigorous and robust method of Colaizzi (1978), as cited by Wirihana et al. (2018:34). This data analysis approach revealed emergent themes and their intertwined relationships (Table 2). The researcher repeatedly read the transcripts and identified significant statements. This led to the extraction of significant statements from the transcripts, which were utilised to formulate meanings. The integration of ideas was to form a cluster of themes. The re-reading of themes ensured the accuracy of findings. Removing redundant descriptions revealed the fundamental structure of advanced psychiatric nurses and prescriptive authority. An invitation was extended to the participants to validate the findings of data analysed and provide additional comments if necessary.

**TABLE 2:** Themes.

Themes	Sub-themes
Prescribing role of the advanced psychiatric nurses	Lack of advanced psychiatric nurses’ utilisation in the prescribing role
Role recognition	The need to have prescribing rights granted

*Source:* Jacobs and Mkhize (2021) unpublished PhD thesis

### Ethical considerations

The Ethics Committee granted ethical clearance (BREC no: 362/18). The gatekeeping permission (NHRD ref: KZ_201808_20) was obtained to conduct the study. There was adherence to the principles of ethical consideration, namely confidentiality, anonymity, and informed consent, throughout the study. The participants were told about their right to withdraw from any stage of the study, if they wish to do so. Permission to record the focus group discussions was also obtained.

## Findings

### Theme 1: Prescribing role of the advanced psychiatric nurses

This theme emerged from the advanced psychiatric nurses in response to the following research question: ‘What are your experiences as advanced psychiatric nurses regarding prescribing’? The participants told that the role of the advanced psychiatric nurse included prescribing medication for mental healthcare users. This theme constitutes the experiences shared by the participants regarding how to fulfil the prescribing role; yet they are not utilised to function in this area. This theme provided insight into what advanced psychiatric nurses have to endure because of the lack of prescribing role utilisation, and their experiences.

#### Sub-theme 1.1: Lack of advanced psychiatric nurses’ utilisation in the prescribing role

The participants expressed frustration, as they were not utilised in the prescribing role. The challenges reported by the participants included the problems that occur during emergency situations when the mental healthcare users need immediate attention, such as serious side effects of their current psychotropic drugs or a need to manage aggressive behaviour. The participants verbalised that they could identify what medication was necessary for a mental healthcare user and the instances in which the dosage was too high and led to side effects. The participants felt despondent because as advanced psychiatric nurses they were unable to perform according to their competencies because of their inability to prescribe medication. The following are extracts from the participants’ statements:

‘“The doctor is so believed to be sole superior in the fraternity of our service. even if the client presents with a simple side effect that we need Disipal, but you can’t just prescribe the Disipal in script”. “The doctor has to do the prescription. Therefore, it means that we have the qualification, but not the power in the clinical setting.”’ (Participant 4, Male, 46 years old)

‘There are certain circumstances whereby, let say that the patient or the client is due for an injection in four days’ time and you can see the life sickness on the patient, but because the doctor is not there, you cannot say that let’s give the injection now and give it now so that he won’t do any damage to himself or others. Because of the fact that I am the nurse, I have to wait for the doctor to do so, or I have to consult with the doctor. Even if the doctor is not available it will mean that we have to wait for the doctor to be available up until a decision can be taken on that matter.’ (P4, Male, 46 years old)

‘“And it feels really bad that with the skill and the knowledge and the exposure that we went through during the training, we cannot come back to the practical setting and apply even those simple things.” We as the nurses, we are working 24/7 with the patients so I feel we know that patients best because after all, even if the doctor comes, we are given the history of the patient and it is so clear at times that you can say this patient is in acute psychosis and needs that injection, but because of our limitations, we cannot, basically, we cannot prescribe, we cannot assess our users and we cannot commence treatment timeously.’ (Participant 5, Female, 56 years old)

‘We need to do the changes on the medication that has been prescribed for the patient and be able to review within such period of time so to tell that if the medication that…uh, … prescribed for the MHCU [*mental health care user*] is working efficiently.’ (Participant 4, Male, 46 years old)

### Theme 2: Role recognition

In this theme, the advanced psychiatric nurses expressed that role recognition to them meant allowing them to prescribe medication for the mental healthcare users in their inpatient institutions. The acknowledgment that this role function could be strengthened by revising legal structures pertaining to prescribing protocols for the advanced psychiatric nurses supported their need to prescribe.

#### Sub-theme 2.1 The need to have prescribing rights granted

According to the participants’ views, prescribing was a skill that advanced psychiatric nurses were well capable of performing; yet they lacked recognition to practise in the role. They raised concern that the advanced psychiatric nurses’ responsibility included conducting mental health state assessments, followed by the necessary prescribing of treatment, which they are unable to do. The participants verbalised that there should be no construing of prescriptive authority as an act to take away the role of the psychiatrist, but rather as a strategy to enhance the need to address the multidisciplinary team approach. The participants told that they believe they have the level of competence and skills to give the desired medication. The need to obtain guidance by the formulation of guidelines would serve as a directive for treatment protocols and establish baselines for advanced psychiatric nurses for what they could and could not do. The following excerpts provide participants’ explanations:

‘Assessing, prescribing. Uhm. Those are the skills that we would like to see and follow what we saw on an assessment like to, you know how to assess and prescribe and to do whatever. And again, when you look at the other PHC [*primary healthcare*] system, let me say, for example, I mean, they got fully entitled right to even use an EDL drug essential, and we cannot!’ (Participant 3, Female, 52 years old)

‘It is not that we are seeing authority to prescribe as a big thing, it’s only that we want our skills to be recognised that we can be able to review and prescribe the medication to the knowledge and skills that we have acquired through our training.’ (Participant 4, Male, 46 years old)

‘I think that guidelines should be prescribed. We should also know our… uh… with regards to work, prescribing, and treating. Perhaps maybe when the guidelines are formulated, limitations, how far can we go. Because at the end of the day we still need to be guided. But we also need that… Uhm. that authority to prescribe within limitations of a job description.’ (Participant 5, Female, 56 yrs)

## Discussion of findings

The advanced psychiatric nurses who participated in the study identified the prescribing role and the need to prescribe medication for mental healthcare users. The advanced mental healthcare nurses shared their experiences in the mental healthcare institutions and described how they felt when they were not granted the permission to prescribe medication.

### Theme 1: Prescribing role of the advanced psychiatric nurses

The prescribing role mentioned in this study refers to the ability of the advanced psychiatric nurses to function to meet the prescribing needs of the mental healthcare users. According to Evans et al. (2020:3), advanced psychiatric nurses have a fundamental role in prescribing in the United Kingdom. They are part of the major contribution made towards the treatment and supportive care offered to patients. Patients benefit from the comprehensive platform of healthcare received, as the nurses can provide holistic care. The view is that the ability to provide comprehensive care autonomously by nurses prescribing is a positive addition to the nurse’s role, as it has enhanced the scope and quality of advanced nursing practice.

Despite the above statement, advanced psychiatric nurses within South Africa are not recognised and not being utilised in the role of prescribing medication for MHCU. As is stipulated within the South African Pharmacy Council (SAPC) (2011:1), nurses are not identified or licensed as authorised prescribers in terms of Section 22A (14) (b). Nurses not being permitted to prescribe is also congruent with the regulatory framework section 56(1) of the *Nursing Act* (Act 33 of 2005), which does not permit prescriptive rights to nurses for schedules 5 and 6 (Jack-Ide, Uys & Middleton 2012:52). This is a clear indication that the closest nurses within South Africa come towards medication modalities in the area of dispensing only. The Essential Drug List (EDL) makes provision for nurses within Primary Healthcare to only prescribe and dispense Schedule 1–4 medicines. Whilst there are existing adult hospital-level Standard Treatment Guidelines (STG), it is apparent they too are only for medical officers and exclude nurses (SA 2019:XXIV).

Countries such as the United Kingdom and America fully embrace the role of advanced psychiatric nurses regarding prescriptive authority compared to South Africa (AANP 2015:1; Hemingway & Ely 2009:24). The view is that the authorisation of nurse practitioners to prescribe would be cost-effective, timely and of quality for MHCU care (AANP 2020:1). The unrestricted prescriptive authority has eradicated the unnecessary limits placed on the nurses in the provision of comprehensive care. In South Africa, the mental health services are undergoing integration into primary healthcare. The move towards integration could be the contributory factor in bringing attention to the need to extend the nurses’ role in the area of prescribing. This means there was consideration given to granting authority for South African nurses to prescribe, which requires them to make use of the Essential Drugs List with limitation to only schedule 1–4 and not psychotropic drugs. The literature provides support that advanced nurses in KwaZulu-Natal, South Africa, have no legal rights to initiate or prescribe medication for mental healthcare users.

Bellaguarda et al. (2015:1067) revealed that whilst there are prescriptive laws recognised for nurses, they still rely and wait upon doctors to validate the prescriptions. This implies that nurses are not able to utilise their skills to practise independently and order medication.

The study revealed that there were still major challenges surrounding the issues of prescriptive authority in South African mental healthcare services. Although advanced psychiatric nurses have acquired the necessary skills and competencies in psychiatric nursing, there is no recognition of prescriptive authority.

### Theme 2: Role recognition

The participants’ need for prescriptive authority requires skills and competence to prescribe psychotropic drugs for the MHCU. In the clinical settings, the advanced psychiatric nurses often face emergencies that warrant immediate medication prescription and administration; yet they are unable to do so. Because of the scarcity of psychiatrists, there is the challenge of waiting for the arrival of the psychiatrist before the implementation of any management for the MHCU. In a study conducted by Klein (2015:163), nurses with advanced training expressed their confidence in the knowledge acquired to practise prescriptive authority. There were no gaps identified due to nurses prescribing medication. Fong et al. (2015:106) stated that various states have adopted different strategies to ensure the adequate preparation and development of advanced nurses for the role of prescriptive authority. The AANP (2020:1) affirmed that nurse practitioners have displayed competence in providing safe prescriptions and of high quality. A study by Bellaguarda et al. (2015:1071) found that prescriptive authority enhances good philosophical grounding, and expert knowledge and competence to prescribe medication.

Numerous states in the United States of America have reconsidered policies and protocols to adapt to the necessary changes in prescriptive authority. Klein (2015:157) acknowledged that notable and substantial changes brought about within the advanced psychiatric nursing roles increased prescribing and medication management for advanced psychiatric nurses. Dong-Lan et al. (2018:72) stated that prescriptive authority was fast growing to ensure effective and proper healthcare delivery in several countries. These authors also identified the benefits of prescriptive authority, which included the influence of dealing with scarcity of doctors, reducing workloads, and meeting the demanding needs for MHCU medication (Dong-Lan et al. 2018:73). However, in South Africa, there is a challenge with the shortage of mental health professionals (especially psychiatrists, psychologists, and advanced psychiatric nurses). The ratio of 0.28% to 100 000 people shows clear evidence that there are insufficient number of mental healthcare professionals to deal with the public mental care system (SA 2019:30). The decreasing output of psychiatrists when compared with the estimation of the population growth makes it unrealistic and practically impossible to be solely dependent on them and to bridge the mental health treatment gap in South Africa. De Kock and Pillay (2017:4) concluded that the implementation of task shifting to address prescriptive gaps, especially within rural settings, is an acceptable practice.

## Conclusion

This study aimed to describe the experiences of the advanced psychiatric nurses regarding prescribing in KwaZulu-Natal, South Africa. From the two themes, namely prescribing role of the advanced psychiatric nurses and role recognition, it became apparent that there were still challenges in fulfilling this role.

Advanced psychiatric nurses are expected to deliver high quality care. But they find themselves unable to use their skills comprehensively as they were not permitted to prescribe medication. Because of the lack of utilisation of advanced psychiatric nurses in KwaZulu-Natal in the prescribing role, they find themselves having to rely fully on the doctors to manage mental healthcare users experiencing psychosis. This draws conclusive findings that the area of prescribing requires attention in KwaZulu-Natal, South Africa, as it appears to be on the back foot of healthcare; international states have progressively engaged in measures to strengthen and enhance this as a fundamental practice. Interestingly, advanced psychiatric nurses in KwaZulu-Natal have no rights to prescribe medication; yet in middle-level income countries, they combat mental healthcare worker shortages by prescribing medication without having proper protocols and prescribing rights. The findings of this article could stimulate further research on prescribing needs in the area of mental healthcare. Moreover, they could identify what protocols could be put in place to ensure that this area becomes recognised.

### Recommendations

The researcher recommended that policy review and protocols in prescriptive authority for advanced psychiatric nurses receive priority. The curriculum review for advanced psychiatric nurses to incorporate the pharmacology module. The prescriptive gaps require investigation to identify and implement protocols within the limited scope of prescribing, whilst waiting for policy changes.

### Limitations

The participants in this study indicated that there was a need for recognition to prescribe medication, but they did not provide information, apart from guideline development, on how to drive this in South Africa.
